# Ethical complexities in contraceptive mandates for adolescent clinical trial participation: a qualitative analysis of U.S. adolescents’ perspectives

**DOI:** 10.1186/s12910-026-01546-9

**Published:** 2026-07-01

**Authors:** Margaret Waltz, Anne Drapkin Lyerly, Hadas Clementine Baron, Rita Vanessa Masese, Suzanne Day

**Affiliations:** 1 Department of Social Medicine, Center for Bioethics, The University of North Carolina at Chapel Hill, 333 South Columbia Street, Chapel Hill, North Carolina 27599 USA; 2 Department of Medicine, Division of Infectious Diseases, The University of North Carolina at Chapel Hill, 130 Mason Farm Road, Chapel Hill, North Carolina 27514 USA

**Keywords:** Adolescents, Clinical trials, HIV, Contraception

## Abstract

**Background:**

Clinical trials often require women and girls of childbearing potential to adhere to contraceptive mandates, typically requiring the use of two forms of contraceptives for the duration of the study. Understanding adolescent perspectives on contraceptive mandates within HIV prevention and treatment trials is critical for evaluating whether and when such requirements are ethically justified and, if so, how they might be appropriately applied in trials involving this group.

**Methods:**

We conducted semi-structured interviews with adolescents age 15–24 in the U.S. on their perspectives about contraceptive requirements. Interviews were audio-recorded and transcribed verbatim. We used Nvivo qualitative analysis software to code transcripts, and then conducted thematic analysis to identify emergent patterns in adolescents’ perceptions of the advantages and disadvantages of requiring contraception use for adolescent participants of HIV research studies.

**Results:**

Forty adolescents were interviewed in total. Overall, most participants expressed support for a contraceptive requirement. Regardless of their stance, perceived advantages and disadvantages of such requirements stood in tension, including the ability to prevent pregnancy and desires to become pregnant, desirable health benefits and concerning side effects, and voluntary participation and violated decisional autonomy.

**Conclusion:**

Our findings illustrate that although contraceptive mandates are intended to facilitate and promote inclusion in research, they can simultaneously work to restrict adolescent participation in research and limit access to its potential benefits. In doing so, these requirements risk undermining their ethical justification and obstructing the generation of data needed to advance health outcomes for adolescents.

## Background

Women’s participation in clinical trials is often conditioned upon adherence to contraceptive mandates, typically requiring the use of two forms of contraceptives for the duration of the study [1, 2]. These requirements are rooted in concerns about preventing fetal harm should pregnancy occur during exposure to investigational products [1, 3, 4]. While such requirements may promote inclusion of women in research by mitigating one aspect of participation risk [1, 5], these mandates have prompted growing ethical concern [4, 6–8]. Some critics warn that the burdens they impose can, at times, outweigh the risks of pregnancy itself [9], and the American College of Obstetricians and Gynecologists’ Committee on Ethics has even described such mandates as “paternalistic, discriminatory, and exclusionary" [8]. Such continued debate begs the question of whether, as a matter of ethics, we should require contraceptives as a condition of women’s participation in the first place and, if so, when.

Contraceptive mandates in clinical trials extend to adolescents, yet there are unique considerations as to the impact of these mandates on adolescents specifically. Adolescents face distinct developmental, social, and health challenges that shape their experiences with both research participation and contraceptive use, including constrained autonomy, limited or inconsistent access to or knowledge about contraceptives, and heightened stigma surrounding sexual activity [10–12]. While adult perspectives on contraceptive requirements in clinical trials have been previously examined [4], adolescent perspectives have been overlooked.

To address this gap, we conducted interviews with adolescents in the United States who have experienced pregnancy and are either living with HIV or at-risk of HIV acquisition. Understanding their perspectives on contraceptive mandates within HIV prevention and treatment trials is critical for evaluating whether and when such requirements are ethically justified and, if so, how they might be appropriately applied in trials involving this group.

## Methods

From May to September 2024, we conducted in-depth interviews to explore adolescents’ perspectives on an array of rules governing their inclusion in HIV prevention and treatment trials, including contraceptive requirements. Individuals were eligible to participate if they were: (1) currently or previously pregnant at any age, and (2) living with HIV and age 15–24, or at-risk of HIV acquisition and age 15–20. The difference in age ranges for eligibility reflects the need to broaden the potential participant pool amidst declines in adolescent HIV incidence in the US [13]. We define ‘at-risk’ for HIV acquisition as having current or prior experience with pregnancy, as pregnancy is both a marker of general risk of HIV acquisition due to sexual exposure and a period associated with increased risk of STI/HIV acquisition [14]. Rather than relying on a legal definition of adolescence (minor vs. adult), we use an expanded definition of adolescence, up to age 24, to account for a broader view of adolescence as a cultural, social, and developmental phase that extends beyond legal cut-offs [15]. This expanded definition is also in keeping with efforts in global HIV research to extend the age range for considering this developmental phase [16].

Adolescents were recruited using study flyers circulated across an adolescent clinical trials research network, a cohort of adolescents participating in a digital HIV-related trial, and local institution-affiliated ob-gyn and infectious disease clinics. Study flyers prompted potential participants to call our study team or complete a Qualtrics form with their contact information. Participants were screened for eligibility and, if eligible, sent the informed consent document. Interviews were conducted one-to-one with a trained interviewer with experience in qualitative research methodology and conducting research with young people (HB); the same interviewer conducted all interviews throughout the study, allowing her to develop insights into how best to frame prompts for participants and anticipate where further clarification might be needed. The interviewer reviewed the informed consent form with the individual, answered any questions, and obtained verbal consent from all participants before the interview. To capture a range of adolescent perspectives on the rules governing their inclusion in HIV research, we obtained a waiver of parental consent for participants who were legal minors in order to remove what has been identified in the literature as a potential barrier to their participation [17, 18]. Given the exploratory purpose of our study and aim to generate in-depth insights rather than thematic saturation, we aimed to recruit a predetermined number of participants (40 in total) per established guidance for information power in qualitative investigations [19]. This study was approved by the University of North Carolina at Chapel Hill Institutional Review Board (protocol #22-2256).

### Data collection

Semi-structured interviews were conducted on Zoom by a trained qualitative interviewer. Questions examined multiple rules characteristically governing adolescents’ participation in HIV prevention and treatment trials, including the requirement to use contraceptives. Using a script (see Table 1), the interviewer described how some biomedical HIV studies require people who can become pregnant to use birth control, often two different forms, with examples of what kinds of contraceptive methods could be used (e.g., pills, condoms, intrauterine device).

**Table 1:** Interview guide description of contraceptive mandates

*Contraceptive Mandate Description*	
I’d like to ask some questions about what rules should be in place for research with adolescents who are pregnant or who might become pregnant. These might be rules from the government, from the hospital or medical center that is running the research, or from the researchers themselves. There are committees (groups of experts) that have to make sure that when research happens it complies with these rules. Some people think that these rules are good, some people think they are not good…	
The [next] rule I’d like to talk about has to do with requiring women and adolescents who could become pregnant to use birth control during a medical study. In some research studies, if a woman or adolescent who is not pregnant wants to join she must agree to use birth control (for example condoms, birth control pills, Depo-Provera injection, or other types of birth control that lasts for several years or more, including a hormonal implant that stops you from getting pregnant for several years, an IUD which is a tiny device put into your uterus that stops you from getting pregnant for several years, or having sterilization surgery to permanently prevent pregnancy). For many studies, they ask she use two types of birth control (for example a condom plus a hormonal implant) for extra protection. If she does not agree to use birth control then she cannot join the study, even if she does not currently have a sexual partner.	

Participants were then asked a series of questions on their perceptions of this rule: whether they felt the rule was good or bad and why, what advantages and disadvantages they saw with this rule, and whether/what impact such a rule might have on their own willingness to participate in biomedical HIV research. Interviews lasted approximately one hour, and each participant received $50 USD for their time.

### Data analysis

Interviews were audio-recorded and professionally transcribed verbatim. Transcripts were reviewed by the study team for accuracy and then coded using NVivo software. The development of the codebook was an iterative and collaborative process. An initial set of parent codes was developed a-priori based on broader topic areas covered in the interview guide, with subcodes subsequently developed inductively through close reading of the transcripts by the team. Three coders team-coded 20% of all transcripts to develop consistency in interpretation and application of codes. The remaining transcripts were individually coded, with a second coder reviewing for fidelity. Notes were made on discrepancies which were resolved through consensus discussion among the coding team. Using a thematic analysis approach [20], the coded data were analyzed to identify emergent themes in participants’ perceptions of contraceptive requirements for adolescent biomedical HIV research participation, producing thematic summaries of key findings. From these summaries, we identified numerous tensions in participants’ views of contraceptive requirements to produce the analysis for this report.

## Results

Forty interviews were conducted, 20 with adolescents living with HIV (LWHIV) and 20 with adolescents at-risk of HIV acquisition (ARHA) (see Table 2). Half (50.0%) of the interviewees were 18–20 years old, 47.5% were aged 21–25, and one participant (2.5%) was aged 15–17. Most participants (67.5%) had completed high school, and the remaining 32.5% had attended college. All interviewees (100%) identified as Christian. Regarding relationship status, 32.5% were partnered but not living together, 25% were single, 25% were married, and 15% were partnered and living together.

**Table 2:** Demographic characteristics of interviewees (*N* = 40)

	**Variable**	**Frequency**	**Percentage**
Age, years	15-17	1	2.5
	18-20	20	50.0
	21-25	19	47.5
Education level	High school	27	67.5
	College	13	32.5
Religion	Christian	40	100.0
Relationship status	Single	10	25.0
	Partnered, not living together	13	32.5
	Partnered, living together	6	15.0
	Married	10	25.0
	Other	1	2.5
Race/ethnicity*	Black	39	97.5
	White	1	2.5
	Other	1	2.5
	Hispanic/Latino	30	75.0
Currently pregnant?	Yes	30	75.0
	No	10	25.0

*Participants could identify with more than one race/ethnicity

Overall, most participants expressed support for the contraceptive rule. Regardless of their stance, participants expressed an array of perceived advantages and disadvantages of the contraceptive rule which often stood in tension, including the ability to prevent pregnancy and desires to become pregnant, desirable health benefits and concerning side effects, and voluntary participation and violated decisional autonomy. These three pairs of tensions are explored in detail below.

### Ability to prevent pregnancy and desires to become pregnant

When asked about any advantages to the contraceptive rule, most participants described pregnancy prevention as a key advantage. Many noted that the rule would help a study participant to “control the number of children she wishes to have” (Participant (P) 1) and prevent “unintended” (P2) or “unwanted” (P3) pregnancies within and outside research studies. Interviewees also saw the rule as providing access to pregnancy prevention that they might not otherwise have if they were not participating in a study. One interviewee said that the rule is “good” because “there are some people that may not even have the information about [birth control], so that would be an exposure to them” (P4). Another similarly said the rule is good “because there are some persons who wouldn’t know how to prevent pregnancies. So, they’re given this opportunity to have this birth control or to use condoms… So, I think it will be helpful for them…” (P5). Some participants said the ability to access contraceptives would make them more likely to participate in research with this mandate, like one who said, “I would be more likely to join… Perhaps they’ll be giving you the condom and pills” (P6). An interviewee who was pregnant said, “I would be more likely to join… At least this should be a reason for me to stay for years without being pregnant… Because I am actually tired of being pregnant. You know, I just want every reason for me not to be pregnant again…” (P7).

Several participants connected the benefit of pregnancy prevention specifically to the research context. One said that being on two forms of birth control is important because it helps ensure “that you don’t get pregnant and then destroy the study” (P8). Another suggested the rule would allow researchers to obtain the data they need by “mak[ing] the girl try to concentrate more and to be a good participant for the research” (P7) in avoiding the competing responsibility of pregnancy. A few interviewees framed the rule as a form of protection during the research process. As one said, “This could be a good rule because it’s totally understandable that…the researcher [is] maybe trying to protect them from…maybe pregnancies or something or being infected or whatever” (P9).

While pregnancy prevention was widely seen as an advantage of the contraceptive rule, this advantage was often in tension with desires to become pregnant. For instance, one participant explained that a mandate would not necessarily be desirable for all adolescents, depending on what is going on in their lives:

[It would] depend on when the study is [conducted]. Maybe someone is just getting married…and is trying to give birth. Like maybe the person hasn’t had her first child yet and that kind of study comes up and people might not like to join depending on the time and depending on what they are trying to do (P10).

For some, extended study periods heightened concerns about the rule’s impact on participants’ reproductive intentions. One interviewee gave an example of a study participant wanting to become pregnant after getting married, saying “maybe for the period of the study, it can be six months, can be three months or thereabouts,” which could be “the months that she might have gotten pregnant. I don’t really think it’s a nice idea” (P11). Another said that “if the study would take a long time, like, maybe a year or two, and the person in question would have to be on this medicine for that long. And maybe the person still wants to give birth, so it may be bad” (P12). Desires to become pregnant shaped some participants’ own reluctance to join a study with a contraceptive requirement. For instance, the same interviewee above said, “for the time that I’m still young and I want to have more children, I would not want to join” (P12). Another similarly mentioned that she would be less likely to join a study requiring contraceptive use “because I got married not so long, and I still want to have children. So, I may not want to join. I don’t know how long the study will take but I will not want to join” (P13).

### Desirable health benefits and concerning side effects

A second prominent advantage of the contraceptive rule that interviewees described was benefits to one’s health in terms of protection against HIV and other STIs. One participant, for instance, explained that the rule is beneficial because “in the case of condoms, it’s going to help to protect you from some sexually transmitted infections” (P14). Another emphasized adolescents’ agency in protecting themselves, saying, “I feel she should be given room to participate if she really wants to…, and then she could actually use the birth control pills or condom to actually protect herself…[from] maybe STD, STI, things like that” (P15). Some participants specifically mentioned HIV prevention as an advantage to the rule. One such interviewee said that the rule could mean someone is “prevented from getting HIV and maybe other diseases,” noting that “early prevention is very good” (P16). Another drew parallels between the benefits of contraceptives and their own use of pre-exposure prophylaxis (PrEP), adding that if someone is on PrEP, “I would say [the rule is] good because…the person is sexually active and also it will prevent the person from getting infected” (P17).

Although participants viewed HIV and STI prevention as a health-related benefit of the contraceptive rule, concerns about potential health risks and side effects associated with mandated contraceptive use were also prominent. One participant said that the rule “might be bad ‘cause of the side effects that [birth control use] might pose because people really react to things differently” (P18). Another elaborated that some birth control “can cause side effects ranging from maybe headache, mood changes, other changes in libido. I think they should be considered because not all types of birth control has worked for people. Also, they should consider some health risk” (P2). A few interviewees also worried about possible impacts on fertility after stopping birth control, including delaying their ability to become pregnant if desired after the study.

These worries about side effects were amplified if participants felt they would not have a choice in which or how many birth control methods to use during the study. For instance, one interviewee said that the rule is bad “only if it has to do with the [birth control] pills. If the pills are going to have the side effects” (P6). Another noted that a disadvantage of the contraceptive rule is “there are some birth controls that have some side effects… All types of birth control may not really go well with everyone. So, I don’t know if the researcher will…do some certain things…to know which kind of birth control is good for each person” (P19). Similarly, an interviewee expressed concerns with using multiple kinds of contraceptives simultaneously, potentially exposing a person to multiple side effects:

I don’t really like the idea of using more than one birth control. I think one should be enough… Some of these things have some side effects… So, I feel that they should know which one would be good for a particular person, and then giving the one [that] makes it…okay for her. (P4)

Ultimately, concerns about side effects influenced desires of some to join studies with a contraceptive rule, as explained by an interviewee who said, “I think there are some people that may be scared of the [contraceptive] drugs because…of the…side effects… So, some people may not use it, may not want to use it, and end up losing or missing the study” (P20).

### Voluntary participation and violated decisional autonomy

A third advantage that emerged from participants’ reflections on contraceptive requirements was the voluntary nature of study participation. Interviewees noted that while contraceptives might be required for participation in a study, people are free to choose not to participate if they do not want to use contraceptives. One participant explained, “I think it’s a good rule… If that is what is to be done, then you should do it that way… I mean if it’s not gonna work with them, then there’s no need for them to participate, right? It just depends on what the researchers need and how they need it to be” (P21). Another echoed this sentiment, saying that “it’s a voluntary activity… So, since you’re not forced to join, it’s free to choose if you want to or not. So, if you feel using birth control is not good for you, then you can stay out of it. But if you’re okay with it, then you join. I’m not seeing anything wrong with that” (P22).

The ability to decline participation if the rule was not agreeable to a potential participant was often cited as being beneficial to the integrity of the research. As one participant said, “The researcher having the participant [use] two kinds of birth control is trying to protect their study from being jeopardized along the line. So, as far as the study, it’s not compulsory. It’s voluntary. So, you have to read the consent form and then agree if you want to participate. So, there’s nothing wrong with that” (P19). Another participant added that those who agree to the rule may be more committed to study participation overall, which would be beneficial to the research: “If the girl refuses to do it, that means they are not interested in the study. Then the people that will do it, that means they’re, let me say, obedient. Yeah. They have adhered to the rules and everything about the study… So, people that decide to join can join. People that refuse, then it’s fine” (P23).

Amidst these benefits to the research, there were also worries that the rule would limit adolescent access to the benefits of research participation. One interviewee, for instance, said that “there are some people that would be willing to join the study, but they don’t want to use the birth control…so they may not be able to join the study and learn what they were supposed to learn and get the information they were supposed to” (P24).

Some participants described the rule as an infringement on their freedom, like a participant who said, “these things are choices that should be made by the individual… You shouldn’t just force someone to do what they don’t want to do or what they don’t feel like doing… Some people may likely not want to take birth control, and some people may likely not want to use the method of birth control” (P25). Similarly, another referenced constraining individual choice, serving as a barrier to participation:

I don’t like the fact that they have to use the birth control to participate. They should be given the free will to, as long as they have the interest, to participate through the study. There shouldn’t be such a rule to participate… Because some persons might not want to use this birth control, but they want to participate in the study. So, imagine if someone doesn’t want to get it, and then there’s a barrier because she has to use it in order to participate (P26).

One participant explicitly framed the issue in terms of autonomy, saying:

Requiring birth control…and making it compulsory before participants can participate in any research…, I see it as…infringing on the participants’ autonomy and their rights to make personal reproductive choices. So, in a situation where I feel I’m being forced into using birth control to participate in a study, I would feel…my voluntary consent is being undermined (P27).

Violation of autonomy was also referenced as a problem for the participation of adolescents for whom contraceptive use might be unacceptable or unnecessary. For example, one participant referenced how cultural and religious beliefs may prohibit contraceptive use, saying, “I wouldn’t say it is all that good because…we have our own personal beliefs, our cultural, and should I say our religious beliefs about birth control. So, I feel in that situation the researchers should be sensitive to all of these personal, cultural, or religious beliefs about birth control” (P28). Others noted that the rule may be unnecessary for those not sexually active, like a participant who said, “agreeing to use the birth control shouldn’t be compulsory. If she doesn’t have a sexual partner, then it’s okay to join the study without a birth control” (P29).

Concerns about limiting decisional autonomy made some less willing to join a study that would require contraceptives. One participant said “I feel like I should make my choices for me. I know what’s best for me. I know what works best for me… I shouldn’t be forced to do what I don’t want to do” (P9). Another participant, who noted the rule would be unnecessary for those not sexually active, said, “if I do not have a sexual partner and the study insists I use a birth control, I would be less likely to join” (P29). Other participants cited their personal beliefs about contraceptives as the reason for opting out or feeling like they “don’t belong” (P27) in a study, like a participant who said, “It’s against my belief. So, making it compulsory that I have to take birth control before I can join any study, already you’re not giving me that option… If I don’t use birth control, I can’t join the study, so definitely I will not join the study” (P30).

## Discussion

Our analysis revealed a trio of tensions surrounding contraceptive mandates for adolescent clinical research. These results mirror prior research with adult women, who similarly cited benefits like access to birth control through trial participation while reporting drawbacks, including a loss of control over one’s reproductive choices and the undue burden these mandates place on women wishing to participate.^4^ That both adolescents and adult women found access to contraceptives as a benefit is striking. In fact, although we had only described the required use of two forms of contraceptives in our interview guide without further details on study specifics, interviewees in this study interpreted contraceptive mandates as beneficial in providing access to contraceptives. We hypothesize that this pattern of benefit perceptions may reflect challenges in obtaining contraceptives and abortion care in the United States – challenges that may be all the more heightened following ongoing legislative changes in abortion access and the reversal of *Roe v. Wade* [21–23]. These growing challenges are especially worrisome for racially and ethnically minoritized groups, particularly Black adults and adolescents who are expected to face the most negative impacts in accessing health care and contraceptives due to new restrictions, are disproportionately located in states with abortion restrictions, and have had lower rates of contraceptive use post-Roe [24–27]. Moreover, difficulties in accessing contraceptives carry distinct ethical implications for adolescents as pregnancy during adolescence is often stigmatized [28], and adolescents face additional barriers to access such as cost, transportation, or worries about confidentiality that further complicate access to reproductive health services [29, 30]. In fact, after the reversal of *Roe v. Wade*, younger people (18–25) were more likely to report difficulties accessing contraceptives than those who are older [22]. Against this backdrop, participation in research with a contraceptive mandate may represent a potentially rare opportunity for adolescents to secure desired contraceptives, a possibility that future research should explore.

However, participants expressed concerns about side effects of contraceptives, the difficulty of participating in research if they wished to become pregnant, and loss of decisional autonomy around contraceptive use. Notably, several participants linked the desire to become pregnant with life transitions such as marriage, suggesting that the timing of studies that conflict with culturally or personally significant milestones for adolescents would make participating in research more difficult. These responses illustrate how contraceptive mandates must be understood within the broader context and complexities of adolescents’ lives and, taken together, reveal a fundamental paradox: although contraceptive mandates are intended to facilitate and promote the inclusion of women and adolescent girls in research, they can simultaneously work to restrict participation in research and limit access to its potential benefits. In doing so, these requirements risk undermining their ethical justification and obstructing the generation of data needed to advance health outcomes for these populations.

Ultimately, this paradox calls for rethinking contraceptive mandates. While such mandates may be warranted in situations where strong preclinical or clinical safety signals indicate that a drug would not be appropriate in pregnancy, their *presumptive* role as appropriate risk management strategies deserves reconsideration in contexts where no such signals exist. In these settings, continued reliance on contraceptive requirements may be difficult to justify and may impose unnecessary burdens on adolescents seeking to participate in research – a population which is already too often excluded from research by default [31]. Indeed, our findings lend support for trial designs centering decision-making about contraceptive use with adolescents, who, as our study suggests, are robustly considering the benefits and harms of contraceptives in relation to their current concerns and priorities. One promising approach is to make contraceptives available as an option rather than a condition of participation, as was implemented in the PURPOSE 1 clinical trial for HIV prevention in young women and adolescent girls [32, 33]. Supporting participants with the informed option to choose whether to use contraceptives respects decisional autonomy while advancing safety and informed choice, an especially important consideration for adolescents given the stigma around their pregnancies and structural barriers to reproductive health services [10–12]. Treating contraceptive use as a decision separate from the decision-making process of research participation also provides opportunities for adolescents to weigh the risks and benefits of each choice independently. Moreover, for interventions likely to be used in pregnancy, this model may produce pregnancy-specific data critical to informed use in clinical settings. Overall, this approach would enable responsible access to trials while generating critical evidence on a population that has been historically underrepresented in research.

These findings should be considered in light of the study’s limitations. Our sample included a limited number of younger adolescents, with many participants at the upper end of the age range of adolescence according to developmental and social parameters [15]. Future research should examine the perspectives of younger adolescents to obtain their views on contraceptives requirements and whether they differ from older adolescents. We also defined “at risk for HIV acquisition” as having current or prior experience with pregnancy. However, pregnancy experience does not capture the full range or variability of HIV acquisition risk, which is shaped by multiple factors, including behaviors, gendered power dynamics, and structural and epidemiological conditions [34–36]. As a result, risk for HIV likely varied across our study population. In addition, our findings may not be generalizable in other global settings as our participants were all based in the United States; while this allows us to consider the important context of our findings regarding the potential impact of national policy shifts, we did not collect location data among our demographic questionnaire, and thus a detailed analysis of state-level factors that may have shaped the perspectives we heard is beyond the scope of this study. Furthermore, while our recruitment strategies were effective in reaching populations disproportionately impacted by the HIV epidemic, the relative homogeneity of the sample in terms of race, ethnicity, and religion should be considered when interpreting and contextualizing these findings. Differences in lived experience, discrimination, and geographical and social contexts may shape perspectives on issues like autonomy, the use of contraceptives, and reproductive decision-making. Given that we did not follow a thematic saturation approach in our data collection, it is also possible that additional themes may arise had we conducted further recruitment. Finally, while participants often spoke about contraceptive requirements in biomedical research broadly, this study was focused on individuals at risk for or living with HIV. Future research should also examine whether similar themes emerge with other populations and groups who may also be important targets for clinical trials.

## Conclusion

Overall, our findings highlight the ethical complexity of contraceptive mandates as well as the nuanced ways adolescents assess potential harms and benefits when deciding whether to participate in a study that requires contraceptive use. Designing trials that respect and support participants’ priorities, and centering decision-making about contraceptives with the research participant, will be critical for fostering adolescent participation, ensuring ethically sound research, and enabling critical evidence generation for a population that has been historically underrepresented in clinical research.

## Data Availability

The de-identified transcribed interviews analyzed during the current study are not publicly available due to ethical and privacy restrictions related to participant confidentiality. The dataset can be requested from the corresponding author upon reasonable request and is subject to institutional approval.

## Abbreviations

LWHIV: Living with HIV
ARHA: At-risk of HIV Acquisition
PrEP: Pre-exposure Prophylaxis
